# A scoping review of pre-hospital technology to assist ambulance personnel with patient diagnosis or stratification during the emergency assessment of suspected stroke

**DOI:** 10.1186/s12873-020-00323-0

**Published:** 2020-04-26

**Authors:** Hannah A Lumley, Darren Flynn, Lisa Shaw, Graham McClelland, Gary A Ford, Phil M White, Christopher I Price

**Affiliations:** 1 Population Health Sciences Institute, Faculty of Medical Sciences, Newcastle University, Newcastle upon Tyne, UK; 2 School of Health and Social Care, Teesside University, Tees Valley, UK; 3 North East Ambulance Service NHS Foundation Trust, Newcastle upon Tyne, England; 4 Medical Sciences Division, Oxford Academic Health Science Network, University of Oxford, and Oxford University Hospitals NHS Foundation Trust, Oxford, UK; 5 Newcastle upon Tyne Hospitals NHS Foundation Trust, Newcastle upon Tyne, England; 6 Northumbria Healthcare NHS Foundation Trust, Newcastle upon Tyne, England

**Keywords:** Stroke, Pre-hospital, Stratification, Diagnosis, Technology, Biomarkers, Imaging, Telemedicine, Ambulance, Paramedic

## Abstract

**Background:**

Pre-hospital identification of key subgroups within the suspected stroke population could reduce delays to emergency treatment. We aimed to identify and describe technology with existing proof of concept for diagnosis or stratification of patients in the pre-hospital setting.

**Methods:**

A systematic electronic search of published literature (from 01/01/2000 to 06/06/2019) was conducted in five bibliographic databases. Two reviewers independently assessed eligibility of studies or study protocols describing diagnostic/stratification tests (portable imaging/biomarkers) or technology facilitating diagnosis/stratification (telemedicine) used by ambulance personnel during the assessment of suspected stroke. Eligible descriptions required use of tests or technology during the actual assessment of suspected stroke to provide information directly to ambulance personnel in the pre-hospital setting. Due to study, intervention and setting heterogeneity there was no attempt at meta-analysis.

**Results:**

2887 articles were screened for eligibility, 19 of which were retained. Blood biomarker studies (*n* = 2) were protocols of prospective diagnostic accuracy studies, one examining purines and the other a panel of known and novel biomarkers for identifying stroke sub-types (versus mimic). No data were yet available on diagnostic accuracy or patient health outcomes. Portable imaging studies (n = 2) reported that an infrared screening device for detecting haemorrhages yielded moderate sensitivity and poor specificity in a small study, whilst a dry-EEG study to detect large vessel occlusion in ischaemic stroke has not yet reported results. Fifteen evaluations of pre-hospital telemedicine were identified (12 observational and 3 controlled comparisons) which all involved transmission of stroke assessment data from the pre-hospital setting to the hospital. Diagnosis was generally comparable with hospital diagnosis and most telemedicine systems reduced time-to-treatment; however, it is unknown whether this time saving translated into more favourable clinical outcomes. Telemedicine systems were deemed acceptable by clinicians.

**Conclusions:**

Pre-hospital technologies to identify clinically important subgroups amongst the suspected stroke population are in development but insufficient evidence precludes recommendations about routine use in the pre-hospital setting. Multi-centre diagnostic accuracy studies and clinical utility trials combining promising technologies are warranted.

## Introduction

Stroke is a medical emergency responsible for a high global burden of mortality and disability, but the outlook is improved by rapid treatment of specific subgroups; such as intravenous thrombolysis for selected ischaemic stroke presentations [1, 2] and mechanical thrombectomy (MT) for large vessel occlusion (LVO) [3]. Due to the increasing centralisation of acute stroke care at specialist facilities [4], early identification and stratification is needed to ensure prompt arrival at the correct hospital for efficient treatment.

For most individuals with acute stroke, the first healthcare contact is a paramedic. However, accurate identification is challenging in the pre-hospital setting due to heterogeneous clinical presentations, time pressure and an absence of simple diagnostic technology. Currently, ambulance personnel use symptom checklists, such as the Face Arm Speech Test (FAST) [5], which have moderate-to-good sensitivity but lower levels of specificity, such that 30–50% of suspected stroke patients later receive an alternative ‘mimic’ diagnosis [5–7]. Once in hospital, stroke and the aetiological sub-type are confirmed via specialist review and brain imaging (computed tomography (CT) and/or magnetic resonance imaging (MRI)) so that mimic cases are excluded and appropriate treatments can be given. In some settings this assessment has been taken to patients using highly equipped ‘mobile stroke unit’ ambulances, with evidence that thrombolysis can be delivered more rapidly [8, 9]. However, current models require a stroke specialist to be present in the vehicle which is not the standard approach for emergency care provision in many healthcare systems.

Currently, key stroke subgroups cannot be identified until after hospital arrival. There is no clinical assessment process which can accurately differentiate between ischaemic and haemorrhagic stroke, and mimics. Now there is overwhelming evidence favouring MT treatment for patients with more severe stroke due to LVO, this group (approximately 10% [10]) should be found as early as possible for direction to those centres able to provide treatment. Symptom severity scores have been developed to assist pre-hospital LVO recognition, but primary evidence of impact on outcome is lacking and accuracy is reduced by mimic, haemorrhagic and non-LVO presentations exhibiting severe symptoms [11, 12].

Pre-hospital assessment based upon symptom checklists alone creates substantial inefficiencies for stroke patients (treatment delays), mimics (displacement to specialist units) and services (additional demands on resources). However, improvement in early stratification/diagnosis of stroke and LVO/ICH subgroups may be possible with emerging technologies which are deployable in standard ambulances [13]. Many are still at early stages of development and will require evaluation of feasibility and resource impact relative to their ability to differentiate, individually or in combination, between key subgroups during the first few hours after symptom onset [14].

### Aim

The aim of this scoping review was to identify, categorise and report the capability of technologies where proof of concept exists for diagnosis and/or stratification of suspected stroke when used by ambulance personnel in the pre-hospital setting.

### Objectives

1. Describe and classify technologies intended to enhance pre-hospital diagnosis of stroke, and/or stratification of suspected stroke, based upon published studies and protocols.

2. Describe evidence for proof of concept and/or feasibility and/or accuracy of the technology according to its stage of development.

3. Describe the impact of the technology on patient care processes or outcomes if data are available.

## Review methods

Methods using standard guidelines [15] have already been described in detail in an online protocol (CRD42018087611) [16].

### Search strategy

Following exploratory searches, with reference to the scoping review question and in collaboration with an information scientist, a systematic search strategy combining MeSH/Web of Science categories and keywords/topic searches was developed and applied to Medline, PubMed, Embase and Web of Science up to 06/06/2019. Hand searching of reference lists and citation searches of included studies were undertaken. Searches were also conducted in online trial registries to identify currently unpublished registered trials on candidate technologies not detected in the literature search. The search strategies are found in 'Additional file 1: Appendix 1'. Papers published before the year 2000 were excluded as there was little emphasis on stroke-specific assessment technologies prior to expansion of the evidence supporting intravenous thrombolysis.

Conference abstracts published after 2013 were identified by applying the search strategy to the databases, with searches limited to ‘conference abstracts’. Grey literature was identified from contact with clinical pre-hospital care content experts. This was done to identify emerging technologies.

### Review criteria

Primary quantitative, qualitative or mixed methods research studies and protocols, including feasibility and pilot studies, with abstracts published in English from any country were eligible for inclusion.

Eligible articles had to describe completed studies or protocols which met the following criteria:

1. (i) Direct diagnostic technologies (e.g. biomarkers) and/or (ii) adjunctive technology to facilitate stratification (e.g. telemedicine requiring equipment which isn’t routinely present in standard ambulances).

2. Application to suspected stroke patients during clinical care.

3. Use by ambulance personnel including paramedics, emergency medical technicians or other clinicians routinely providing pre-hospital care in specific countries, e.g. EMS physicians in Germany [17, 18].

4. How the result would be available to EMS clinicians in the pre-hospital setting (prior to hospital admission).

5. Technology at any stage of development as long as it was (or protocols where it is planned to be) applied during ambulance care of suspected stroke.

Ambulance-based studies were excluded if the technology would not be transferrable to standard ambulances without them becoming specialised vehicles (e.g. Mobile Stroke Units which include Computed Tomography (CT) and a Point of Care lab). Reports which described paper-based algorithms, clinical rules and clinical scales, studies involving medical personnel not routinely present in the pre-hospital setting, hospital-based studies, studies focused on paramedic training and studies which did not exclusively include populations with suspected stroke were also excluded.

### Study selection

Two reviewers (HL and DF) independently assessed titles/abstracts (stage 1). The same two reviewers independently assessed full texts of retained studies for their conformity to the inclusion criteria using a study selection form. Disagreements at the second stage were resolved via discussion or adjudicated by a third reviewer (CP).

### Data extraction

Data were independently extracted by two reviewers (HL and DF), with discrepancies solved via discussion or adjudication (CP). A data extraction form captured detailed information on studies and technologies (Additional file 1: Appendix 2). As no adequate framework to evaluate diagnostic technology studies existed for this purpose, a modified version of the TIDieR framework [19] was used to standardise descriptions. This included generic study information (e.g. authors; publication year, country, purpose [diagnostic and/or facilitation of care], research design and key findings) and detailed information about the technologies. As the scope of the review was worldwide, we did not use the European Union CE mark guidance on physical invasiveness, and a simpler classification system was used [20]: invasive (penetration/breaking of the skin or entry into a body cavity); minimally invasive (indirect observation of internal areas of the body); and non-invasive (no penetration or breaking of the skin). The stage of development was recorded as alpha (initial prototype stage), beta (later iteration of the prototype, feature complete but not finalised) or gamma (finalised product available for wider use) [21].

### Data synthesis

As this was a scoping review, there was no a-priori plan for data meta-analysis. Extracted information was used to develop a taxonomy describing direct or facilitative stratification technologies according to their mode of action. Where reported, data on true/false positives and negatives were used to calculate sensitivity and specificity values. Studies were classified according to study design to inform recommendations based upon strength of evidence.

## Results

**Fig. 1:**
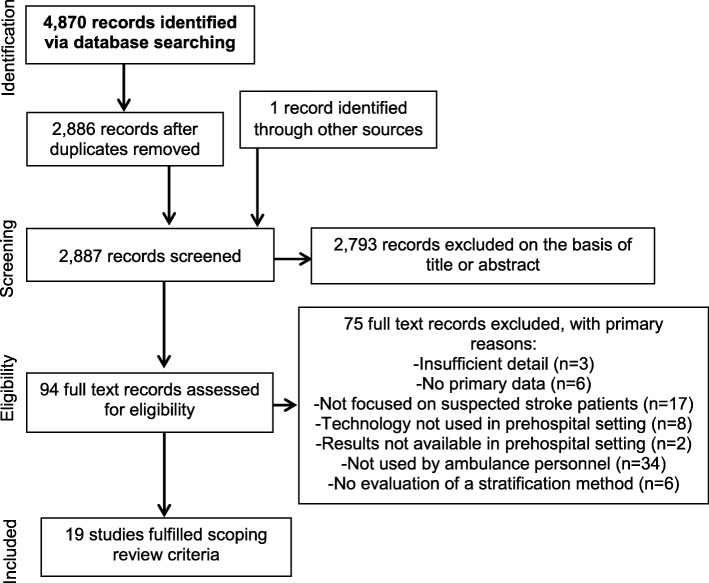
Flow diagram summarising the process used to identify studies

A total of 4870 (2886 after deduplication) studies were identified via the search strategy (Fig. 1). One study was identified via citation searches. 94 potentially eligible articles were retained following initial screening. After obtaining and reviewing full texts (or abstracts where full texts were not available), 75 studies were excluded with reasons detailed in Fig. 1. In total, 19 studies were included, 8 of which were full text articles, 7 conference abstracts and 4 published protocols.

### Description of included studies

Technologies employed by included studies [22–40] were best described using three categories: blood biomarkers [22, 23]; pre-hospital imaging [24, 25] and mobile telemedicine/telestroke (including transfer of audio and/or visual information or relevant data) [26–40]. These are summarised in Table 1.

**Table 1:** Characteristics of included studies

Category	Product / Project Name	Author/Year	Title	Country	Article type	Enrolled patients	Age (median) & Gender	Time window	Development stage	Study Design
Biomarkers	Helsinki Ultra-acute Stroke Biomarker Study	Lindsberg 2018 [22]	Helsinki Ultra-acute Stroke Biomarker Study	Finland	Protocol	N/A	N/A	Ultra-acute	Alpha	Protocol:Single-centre Prospective diagnostic accuracy
Biomarkers	SMARTChip / PRISM	Shaw et al. 2019 [23]	Purines for Rapid Identification of Stroke Mimics (PRISM)	UK	Protocol	N/A	N/A	NR	Alpha	Protocol: Multicentre Prospective diagnostic accuracy
Prehospital imaging	Handheld Infra-red Screening Device	Murphy et al. 2015 [24]	Measurement of acute brain hemorrhage in the pre-hospital setting	USA	Results	46 suspected stroke	NR	NR	NR	Single-centre Prospective diagnostic accuracy
Prehospital imaging	ELECTRA-STROKE	Coutinho 2019 [25]	Electra-Stroke:EEG controlled triage in the ambulance for acute ischemic stroke	Netherlands	Protocol	N/A	N/A	Acute	Beta	Protocol: Multicentre Prospective diagnostic accuracy
Telestroke	TeleBAT	LaMonte et al. 2000 [26]	TeleBAT: Mobile telemedicine for the brain attack team	USA	Results	6 suspected stroke	NR	NR	Beta	Single-centre Development and pilot test
		Xiao et al. 2000 [27]	Design and evaluation of a real-time mobile telemedicine system for ambulance transport	USA	Results		NR	NR		
Telestroke	Med-on-@ix /‘peeq-box’	Bergrath et al. 2012 [28]	Feasibility of pre-hospital teleconsultation in acute stroke: a pilot study in clinical routine	Germany	Results	64 suspected stroke	80 years 64% female	Acute	Beta	Multicentre Prospective non-randomised trial
Telestroke	Stroke Angel	Ziegler et al. 2008 [29]	Mobile computing systems in preclinical care of stroke: Results of the stroke angel initiative within the BMBF project PerCoMed	Germany	Results	443 suspected stroke	NR	Acute	Beta	Multicentre Prospective cohort
		Rashid et al. 2015 [30]	Stroke angel: Telemedicine prenotification improves door-to-CT and rate of systemic thrombolysis	Germany	Results	1262 suspected stroke	NR	Ultra-acute	Gamma	Single-centre Prospective cohort
Telestroke	PreSSUB I	Espinoza et al. 2016 [31]	Development and Pilot Testing of 24/7 In-Ambulance Telemedicine for Acute Stroke	Belgium	Results	16 suspected stroke	NR	Acute	Beta	Single-centre Development and pilot test
Telestroke	PreSSUB II	Espinoza et al. 2015 [32]	PreSSUB II: The prehospital stroke study at the Universitair Ziekenhuis Brussel II	Belgium	Protocol	N/A	N/A	Acute	Beta	Protocol: Single-centre RCT
		Brouns et al. 2016 [33]	24/7 In-Ambulance telestroke: Results from the pre-hospital stroke study at the universitair ziekenhuis brussel II (PreSSUB II)	Belgium	Results	1 suspected stroke	NR	Acute	Gamma	Single-centre RCT
Telestroke	InTouch Xpress	Belt et al. 2016 [34]	In-transit telemedicine speeds ischemic stroke treatment: preliminary results	USA	Results	163 suspected stroke	NR	Ultra-acute	Gamma	Multicentre Feasibility and pilot test
Telestroke	Smartphone with encrypted software	Brotons et al. 2016 [35]	The use of prehospital telemedicine to aid in the decision to airlift patients to a comprehensive stroke center from a rural area.	USA	Results	45 suspected stroke	NR	NR	Beta	Single-centre Feasibility and pilot test
Telestroke	HipaaBridge with iPads	Barrett et al. 2017 [36]	Ambulance-based assessment of NIH Stroke Scale with telemedicine: A feasibility pilot study	USA	Results	11 suspected stroke	65 years 81% female	NR	Beta	Single-centre Feasibility and pilot test
Telestroke	iPad with video capability	Shah et al. 2017 [37]	A novel use of out-of-hospital telemedicine to decrease door-to-computed tomography results in acute strokes	USA	Results	NR	NR	NR	Beta	Single-centre Prospective cohort
Telestroke	Field-Telestroke	Andrefsky et al. 2018 [38]	Impact of EMS field-telestroke with hand-held iPads on IV-TPA therapy for stroke	USA	Results	NR	NR	Ultra-acute	Beta	Single-centre Retrospective before and after
Telestroke	REACHOUT	Hackett et al. 2018 [39]	EMS based telestroke suggests reduced door to needle time compared to hospital based telestroke	USA	Results	200 suspected stroke	NR	Ultra-acute	Beta	Multicentre Prospective cohort
Telestroke	Custom-built system	Johansson 2019 [40]	Technical feasibility and ambulance nurses’ view of a digital telemedicine system in pre-hospital stroke care: a pilot study	Sweden	Results	11 suspected stroke	NR	NR	Beta	Single-centre Mixed methods pilot test

Most studies were conducted in the USA [24, 26, 27, 34–39], followed by Germany [28–30] and Belgium [31–33]. One study was conducted in each of the following countries: UK [23], Finland [22], Netherlands [25] and Sweden [40].

### Blood biomarkers

We identified protocols for two currently active, large pre-hospital studies of blood biomarker technologies which are both prospective diagnostic accuracy designs [22, 23], summarised in Table 2.

**Table 2:** Descriptions of Biomarker Technologies and Outcomes

Study Details	Portability	Expertise and training requirements	Person interpreting output	Location (on scene; stationary ambulance; in transit)	Purpose, diagnostic accuracy, comparator and use of clinical scale	Physical invasiveness and time to acquire results	Acceptability: clinicians and/or patients	Impact on EMS clinician decisions or treatment provision	Impact on process (time metrics) or patient outcomes	Costs
**Helsinki Ultra-acute Stroke Biomarker Study** Lindsberg 2018 [22] Sub-type: Proteins	Portable with use of a vehicle	**Expertise:** EMS clinician **Training:** not reported	EMS clinician	In transit	**Purpose:** Diagnostic -Mimic -TIA -Ischaemia -Haemorrhage **Diagnostic accuracy:** Results not yet available **Comparator:** Definitive diagnosis **Clinical Scale:** none	Invasive **Time to acquire results:** not yet available	Not assessed	**Destination:** Results not yet available **Treatment:** Results not yet available	**Time metrics:** Not yet available **Patient outcomes:** will be evaluated using 3-month modified Rankin Scale (mRS)	Not reported
**Purines for Rapid Identification of Stroke Mimics (PRISM)** Shaw et al. 2019 [23] Sub-type: Purines (Metabolite)	Highly portable (hand-held)	**Expertise:** EMS clinician **Training:** Required	EMS clinician	On scene Stationary In transit	**Purpose:** Diagnostic -Mimic -TIA -Ischaemia -Haemorrhage **Diagnostic accuracy:** Results not yet available **Comparator:** Expert clinical opinion informed by brain imaging and/or other investigations **Clinical scale:** FAST	Invasive **Time to acquire results:** ~ 3–5 min	Results not yet available	**Destination:** Results not yet available **Treatment:** Results not yet available	Not yet available	Not reported

The Helsinki Ultra-acute Stroke Biomarker Study [22] is an early stage, single-centre study aiming to establish diagnostic and predictive biomarkers for potential IVT candidates. The specific aims are to (i) identify ischemic stroke, transient ischemic attack, intracerebral haemorrhage and stroke mimics; (ii) identify patients not responding to IVT; (iii) identify patients with increased chance of IVT-related complications; and (iv) predict 90-day patient health outcomes using the modified Rankin Scale (mRS). The study will evaluate known stroke biomarkers, e.g. Glial Fibrillary Acidic Protein (GFAP) and NR2 peptide, and explore novel markers (during a discovery phase) via blood samples taken by EMS clinicians during transit. This study has yet to report on primary outcomes, although a precursor study [41] has reported the feasibility of implementing pre-hospital EMS biomarker sampling using a cannula adapter technique. It is not clear how EMS clinicians would obtain a result on scene in future (e.g. a point of care assay system) even if the panel is of value.

Purines for Rapid Identification of Stroke Mimics (PRISM) [23] is an early stage, multi-centre study aiming to assess the diagnostic accuracy of whole blood purine concentration using capillary blood sampling performed by trained EMS clinicians within 4 h of stroke symptom onset. The goal is to differentiate between stroke and stroke mimics, with a hospital sub-study investigating LVO. The point-of-care SMARTChip system, a hand-held reader and disposable biosensor, developed by Sarissa Biomedical Ltd. in the UK, measures purines in a finger-prick blood sample. Purines are a by-product of cellular metabolism which accumulate rapidly during hypoxia (as occurs in stroke) and can be reliably detected in systemic arterial blood [42]. Results can be obtained within 3–5 min and paramedics require training to use this technology [43]. There is currently no published diagnostic accuracy or patient outcome data, however the technology is at a late stage of development which would facilitate deployment if of value. Neither biomarker protocol commented on the potential cost of the technologies.

### Pre-hospital imaging

We identified two studies of non-invasive pre-hospital imaging technology [24, 25]. These are summarised in Table 3. EEG is included here as ‘imaging’ since the intention is to produce information which correlates anatomically with cerebral tissue injury. From a study design perspective, these are prospective diagnostic accuracy studies.

**Table 3:** Descriptions of Pre-hospital Imaging Technologies and Outcomes

Study Details	Portability and Resolution	Expertise and training requirements	Person interpreting output	Location (on scene; stationary ambulance; in transit)	Purpose, Diagnostic accuracy, comparator and clinical scale	Physical invasiveness and time to acquire results	Acceptability: clinicians and/or patients	Impact on EMS clinician decisions or treatment provision	Impact on process (time metrics) or patient outcomes	Costs
**“Handheld Infra-red screening device”** Murphy et al. 2015 [24] Electromagnetic Spectroscopy – Light FDA approved CE Marked ISO certificatio	**Portability**: Highly portable (hand-held) **Resolution:** Low resolution	**Expertise:** Qualified Paramedic **Training:** required	EMS clinician	In transit	**Purpose:** Diagnostic -Stroke -Mimic -Haemorrhage **Diagnostic accuracy:** **Haemorrhage vs Mimic** False Positive Rate: 15 False Negative Rate: 2 True Positive Rate: 5 True Negative Rate: 10 *Extrapolated Values*: Sensitivity: 71% Specificity: 40% **Haemorrhage vs Ischaemia** False Positive Rate: 8 False Negative Rate: 2 True Positive Rate: 5 True Negative Rate: 6 *Extrapolated Values*: Sensitivity: 71% Specificity: 42% **Comparator:** CT (hospital) scan **Clinical scale:** stroke assessment form	Non-invasive **Time to acquire results:** 2–3 min	**Clinicians:** 2–3 min too long (+increased FPR if time reduced). Measurements performed during fast ambulance transport appeared to cause false positive readings Unreliable measurement for combative and epileptic patients.	**Destination:** None **Treatment:** Not reported	Not reported	Not reported
**Electra-Stroke** Coutinho 2019 [25] EEG Waveguard™ dry electrode cap and the eego™ amplifier are both CE marked	**Portability:** Portable with use of a vehicle **Resolution:** Low Resolution	**Expertise:** Qualified paramedic **Training:** Not reported	Unclear	In transit Other locations unclear	**Purpose:** Diagnostic -LVO-a **Diagnostic accuracy:** Results not yet available **Comparator:** Final (hospital) diagnosis **Clinical scale:** mRS, NIHSS	Non-invasive **Time to acquire results:** ‘Under 5 min’	Results not yet available	**Destination:** Results not yet available **Treatment:** Results not yet available	Results not yet available	Not reported

Our review identified one small, single-centre study reporting on use of a handheld Infrared screening device in the pre-hospital setting in the USA by device-trained EMS clinicians during transit [24]. The stage of development was not reported. The purpose of the device was to discern between stroke types by detecting changes in blood flow. The authors found evidence that contralesional increases in blood flow indicate LVO. The device was compared with hospital-based CT on its ability to detect haemorrhagic stroke in 46 suspected stroke patients; 7 of these 46 patients had CT-confirmed haemorrhagic stroke. True and false positive/negative results are shown in Table 3; diagnostic accuracy was extrapolated from these figures. For haemorrhage versus mimic, sensitivity and specificity within the study population were 71 and 40% respectively. Sensitivity and specificity for haemorrhage versus ischaemia were 71 and 43% respectively. Although all haemorrhagic strokes were identified by the device, the poor specificity may limit clinical use. Paramedics considered the scan too long for stroke patients and consequent speeding up of the patient scan time increased false positives. Faster ambulance speed also increased false positive rates, and the device was difficult to use reliably with patients unable to lie still. There was no impact on destination decisions or reported change in patient outcomes.

‘Dry’ electrode cap Electroencephalography (EEG) will be used in the ELECTRA-STROKE study [25], which is a large, active multi-centre study in the Netherlands, aiming to develop and validate an algorithm for automated signal analysis to detect anterior circulation LVO in suspected ischaemic stroke patients in the pre-hospital setting. The technology is fully developed; however, development of the algorithm is in early stages. The rationale for this study is underpinned by evidence that delta activity is associated with lesion location on cerebral imaging [44]. Omitting the preparation time for ‘wet’ EEG may enable even inexperienced EMS clinicians to undertake a measurement within 5 min. Training requirements were not reported. Across 4 phases, algorithms will be iteratively tested and developed to maximise diagnostic accuracy using the CE-marked Waveguard™ dry electrode cap and the eego™ amplifier. The algorithm will be validated in the ambulance in a large multi-centre study. The EEG results will only be analysed in hospital so destination choice will not be assessed. It is not reported whether clinical outcomes will be assessed. Expected primary outcome completion was December 2019.

There are no data available on costs, EMS clinician decisions regarding hospital destination (stroke-specialist versus non-stroke specialist centre), impact on treatment or patient outcomes for either pre-hospital imaging device.

### Mobile telemedicine

We identified 12 mobile telemedicine technologies reported across 15 studies [26–40]. Descriptions are summarised in Table 4. The outcomes are summarised in Table 5. Eleven studies were single-centre [26, 27, 30–33, 35–38, 40], one of which was large [30], and four were moderate-to-large multi-centre studies [28, 29, 34, 39]. From a design perspective, this included: development and pilot testing (*n* = 2), feasibility and pilot testing (*n* = 3), mixed methods pilot test (*n* = 1), retrospective before and after (*n* = 1), prospective cohort (*n* = 4), a prospective non-randomised controlled trial (*n* = 1), protocol: single-centre RCT (*n* = 1), single-centre RCT (*n* = 1).

**Table 4:** Descriptions of Telestroke Technologies

Study Details	Communication Method	Portability, Resolution and Data Transfer Speed	Expertise and training Requirements	Person interpreting output	Location (on scene; stationary ambulance; in transit)	Costs
**TeleBAT** LaMonte et al. 2000 [26] Xiao et al. 2000 [27]	Unidirectional video and data transfer	Portable with use of a vehicle **Resolution:** Low (VGA) **Transmission speed:** Slow (2G)	**Expertise:** Qualified Paramedic **Training**: required	Remote physician	Stationary In transit	$20,000–$25,000 (~£14,000 - £17,000) in year 2000 + ‘operating cost of 4 cell phones’
**‘peeq-box’** Bergrath et al. 2012 [28]	Bidirectional video and data transfer	Portable with use of a vehicle **Resolution:** Low (VGA) **Transmission speed:** Slow to moderate (2G–3G)	**Expertise:** Other ambulance staff (EMS physicians) **Training**: required	Remote physician	Stationary In transit	Not reported
**Stroke Angel** Ziegler et al. 2008 [29] Rashid et al. 2015 [30]	Clinical data transfer No audio or video	Highly portable (hand-held) **Resolution:** N/A **Transmission speed:** Slow (2G) [29] to moderate (3G) [30]	**Expertise:** Qualified Paramedic **Training:** required	EMS clinician	Stationary In transit	Not reported
**PreSSUB I** Espinoza et al. 2016 [31]	Bidirectional video and data transfer	Portable with use of a vehicle **Resolution:** High (HD-FHD) **Transmission speed:** Fast (4G)	**Expertise:** Other ambulance staff (EMS nurses) **Training:** required	Remote physician	In transit	Not reported
**PreSSUB II** Espinoza et al. 2015 [32] Brouns et al. 2016 [33]	Bidirectional video and data transfer	Portable with use of a vehicle **Resolution:** (HD-FHD) **Transmission speed:** Moderate to fast (3G–4G)	**Expertise:** Other ambulance staff (EMS nurses) **Training:** required	Remote physician	In transit	Not reported
**InTouch Xpress** Belt et al. 2016 [34]	Bidirectional video	Highly portable (hand-held) **Resolution:** High (HD-FHD) **Transmission speed:** Fast (4G)	**Expertise:** Qualified Paramedic **Training:** required	Remote physician	In transit	~$33,000 (~£27,000) in 2016: $23,000 (~£19,000) equipment + ~ $10,000 (~£8000) maintenance
**Smartphone with encrypted software** Brotons et al. 2016 [35]	Bidirectional video (unclear if data transfer)	Highly portable (hand-held) **Resolution:** Not reported **Transmission speed:** Not reported	**Expertise:** Qualified Paramedic **Training:** not reported	Remote physician	On scene Stationary	$2250 (~£1800) per unit in 2016
**HipaaBridge** Barrett et al. 2017 [36]	Bidirectional video	Highly portable (hand-held) **Resolution:** High (HD-FHD) **Transmission speed:** Fast (4G)	**Expertise:** Qualified Paramedic **Training**: required	Remote physician	In transit	~$600 (~£500) in 2017
**iPad with video capability** Shah et al. 2017 [37]	Bidirectional video	Highly portable (hand-held) **Resolution:** Not reported **Transmission speed:** Not reported	**Expertise:** Qualified Paramedic **Training**: required	Remote physician	Not reported	Not reported
**Field-Telestroke** Andrefsky et al. 2018 [38]	Bidirectional video	Highly portable (hand-held) **Resolution:** Not reported **Transmission speed:** Not reported	**Expertise:** Qualified Paramedic **Training**: required	Remote physician	On scene In transit	Described as ‘Low cost’
**REACHOUT project / HIPPA-compliant hand-held iPads** Hackett et al. 2018 [39]	Bidirectional video	Highly portable (hand-held) **Resolution:** Not reported **Transmission speed:** Not reported	**Expertise:** Qualified Paramedic **Training**: requirements were not reported	Remote physician	Not reported	Not reported
**Custom-built system** Johansson et al. 2019 [40]	Bidirectional video	Portable with use of a vehicle **Resolution:** High (HD-FHD) **Transmission speed:** Moderate to fast (3G–4G)	**Expertise:** Other ambulance staff (EMS nurses) **Training:** required	Remote physician	Not reported	Not reported

**Table 5:** Telestroke Technology Study Outcomes

Study details	Purpose, Diagnostic accuracy, comparator and clinical scale	Time to conduct telestroke assessment	Acceptability: clinicians and/or patients	Impact on EMS clinician decisions or treatment	Impact on process (time metrics) outcomes	Impact on patient outcomes
**TeleBAT** LaMonte et al. 2000 [26] Xiao et al. 1997 [27]	**Purpose:** Stratify stroke/facilitate care **Diagnostic accuracy:** Not reported **Comparator:** None. Assessed acceptability and usability of TeleBAT **Clinical scale:** NIHSS	Not reported	**Paramedics × 2 and stroke specialists × 2** [26]: Clinicians in favour of TeleBAT (privacy of video transmission, non-interference with regular tasks on ambulances; providing valuable information; & usability) **Paramedics × 2 and stroke specialists × 2**[27]: System did not intrude into paramedic/patient privacy and was safe. Adequate for clinical examinations: stroke specialists could score most NIHSS items, but difficulty with patients’ leg movement). Easy to learn/operate.	**Destination:** Not reported **Treatment:** Not reported	Not reported	Not reported
**‘peeq-box’** Bergrath et al. 2012 [28]	**Purpose:** Stratify stroke/facilitate care **Diagnostic accuracy:** prehospital stroke diagnosis confirmed in hospital in 11 patients (61%, telestroke) vs 30 (67%, standard EMS) – difference non-significant *Extrapolated data:* Telestroke: False Positive Rate: 7; True Positive Rate: 11 vs standard EMS transport False Positive Rate: 15; True Positive Rate: 30 Non-significant differences between telestroke and standard EMS for other neurological/non-neurological diagnoses **Comparator:** Standard EMS transport (time metrics) and hospital-confirmed diagnosis **Clinical scale:** bespoke 14-item stroke history checklist + Glasgow Coma Scale	Not reported	In 15 of 18 missions the telemedicine system functioned faultlessly. Significantly more (median 14) stroke-specific data points were transferred, in written form, from the EMS to the hospital via telestroke (versus median of 5 non-telestroke group).	**Destination:** Not reported **Treatment:** No significant impact on thrombolysis rates: 3/10 (30%) telestroke 5/27 (19%) standard EMS	**Sample of patients with a suspected pre-hospital diagnosis of stroke** **Time on Scene:** 4 min median increase with Telestroke (median 25 mins) vs standard EMS (median 21 mins). Difference was non-significant **Scene to door time:** 2.5 min median increase with Telestroke (median 37.5 mins) vs standard EMS (median 35 mins). Difference was non-significant **Door-to-scan time:** 2 min increase with Telestroke (median 59.5 mins) vs standard EMS (median 57.5 mins. Difference was non- significant	Not reported
**Stroke Angel** Ziegler et al. 2008 [29]	**Purpose:** Stratify stroke/facilitate care **Diagnostic accuracy:** *Extrapolated data:* Stroke vs non-stroke False Positive Rate: 27 False Negative Rate: 53 True Positive Rate: 102 True Negative Rate: 44 Sensitivity = 65.81% Specificity = 61.97% **Comparator:** Hospital-based assessment using the same clinical scales and changes in time metrics before (prior to 2005) and after (2005–2007) introduction of Stroke Angel **Clinical scales:** Los Angeles Prehospital Stroke Screen, 3-item stroke scale	Not reported	Benefits stated by hospital clinicians were that EMS clinicians are “trained” by direct feedback from the PDA in dealing with the stroke patient. The use of Stroke Angel was evaluated to be consistently positive by EMS clinicians. Hospital clinicians took the early warning seriously and were better prepared for the arrival of patients. Better communication between doctors and EMS clinicians, and improved perception of each other’s tasks and work.	**Destination:** Not reported **Treatment:** Local lysis rate (number of lyses / all stroke patients enrolled on the stroke unit) increased from 6.1% (2005) to 11.2% (2007)	**Call to scene time**: unchanged (10 mins before and after) **Time on scene:** before (17 mins); after (2007) 23 mins **Travel time:** before (26 mins) after (2007) 22 mins **Call to-door time:** before (53 mins); after (2007) 55 mins **Door-to-CT time:** before (53 mins); after (2007) 30 mins *Patients treated with thrombolysis:* **Door to CT time:** before (32 mins); after (2007) 16 mins **Door-to-needle time:** before 61 mins, after (2007) 38 mins	1.5% of cases with symptomatic intracerebral bleeding (SITS-MOST criteria)
**Stroke Angel** Rashid et al. 2015 [30]	**Purpose:** Stratify stroke/facilitate care **Diagnostic accuracy:** Not reported **Comparator:** Standard EMS transport (time) **Clinical scale:** ‘Structured checklist’	Not reported	Not reported	**Destination:** Not reported **Treatment:** Telestroke (39%), standard EMS (32%). 7% difference statistically significant	**Data covered the period 2005–2013:** **Time on scene:** 19 mins (Telestroke), 20 mins (Standard EMS). Not statistically significant **Door-to-scan time:** 12 mins (Telestroke), 24 mins (Standard EMS). Difference of 12 mins was statistically significant	Not reported
**PreSSUB I** Espinoza et al. 2016 [31] Certified by Autographe (Wavre, Belgium)	**Purpose:** Stratify stroke/facilitate care **Diagnostic accuracy: T**eleconsultants identified 12 patients (80%) with potential stroke or TIA, which concorded with in -hospital diagnosis in 10 patients (83%). Telestroke – no missed stroke diagnoses: *Extrapolated data:* False positives: 2; False negatives: 0; True positives: 10 **Comparator:** Hospital-based diagnosis **Clinical scale:** Unassisted Telestroke Scale	Median 9 min (IQR 8–13 min)	NIHSS was considered unsuitable for mobile telemedicine – this led to the development of a novel scale to rapidly assess stroke severity via telemedicine without assistance by a third party – the Unassisted Telestroke Scale. 94% of teleconsultations were established successfully; one major technical issue occurred due to battery malfunction of the in-ambulance device.	**Destination:** Not reported **Treatment:** Not reported	Not reported	Not reported
**PreSSUB II** Espinoza et al. 2015 [32] Brouns et al. 2016 [33] Certified by Autographe	**Purpose:** Stratify stroke/facilitate care **Diagnostic accuracy:** Not reported **Comparator:** Standard EMS transport (time) and hospital diagnosis **Clinical scale:** Glasgow Coma Scale, Unassisted Telestroke Scale (UTSS)	Not reported	The proportion of successful in-ambulance telemedicine assessments was 96.2% [33]. Technical and organisational feasibility was established [33].	**Destination:** Not reported **Treatment:** Thrombolysis rate (not yet available)	**Call-to-CT time** [33]**:** Standard EMS (87.1 min; 95% CI = 68.7–105.6) versus telestroke (50.8 min; 95% CI = 46.3–55.3): Statistically significant mean reduction of 36.4 min (95% CI = 17.5 to 55.3)	No telestroke-related adverse events. Mortality was similar in both groups [33] mRS, Barthel Index, EQ-5D and WHO-Five Well-being Index (not yet available)
**InTouch Xpress** Belt et al. 2016 [34]	**Purpose:** Stratify stroke/facilitate diagnosis: -Stroke -Ischemia **Diagnostic accuracy:***Extrapolated data:* Stroke vs non-stroke (telestroke) False Positives: 3 True Positives: 12 Stroke vs non-stroke (Standard EMS transport) False Positives: 17 True Positives: 54 **Comparator:** Standard EMS transport (time) and hospital diagnosis. **Clinical scale:** Cincinnati Stroke Scale	With alteplase (*n* = 15): mean 7.3 mins (95% CI = 4.9–9.8). Without alteplase (*n* = 74): mean 4.7 mins (95% CI = 3.9–5.4)	Clinicians: 39% of teleconsults required reconnection. Connectivity was rapidly re-established in all but two cases; in all but these two cases, the tele-neurologist felt the clinical evaluation was satisfactory. Acceptance among patients and EMS has been uniformly positive (but no data are presented to support this statement).	**Destination:** Not assessed **Treatment:** Not reported	**Door to needle time:** Telestroke - mean 28 mins Standard EMS – mean 41 mins (decrease of 13 min was statistically significant) **Onset to scene time:** Telestroke - mean 31.1 mins Standard EMS – mean 50 mins (18.9 min decrease was non-significant) **Scene-to-door time:** Telestroke - mean 29 mins Standard EMS – mean 34 mins (5 min decrease was non-significant) **Onset to needle time:** Telestroke - mean 92 mins Standard EMS – mean 122 mins (32 min decrease was significant)	Deaths: 0 (in both groups) Complications: 1 in telestroke group (vs 5 in standard EMS group)
**Smartphone with encrypted software** Brotons et al. 2016 [35]	**Purpose:** Stratify stroke/facilitate care **Diagnostic accuracy:** 'High correlations' between telestroke NIHSS and NIHSS on hospital arrival **Comparator:** Telestroke NIHSS versus arrival at hospital NIHSS (conducted by the same physician) **Clinical scales:** CPSS, MEND exam	Not reported	Paramedics and physicians: easy to use and extremely valuable in making triage decision.	**Destination:** Direct transfer to CSC **Treatment:** Not reported	Not reported	Not reported
**HipaaBridge on iPads** Barrett et al. 2017 [36]	**Purpose:** Stratify stroke/facilitate care **Diagnostic accuracy:** Not reported **Comparator:** None. Assessed acceptability and usability of HipaaBridge **Clinical scales:** NIHSS	Mean NIHSS assessment time 7.6 mins (range 3 to 9.8 mins)	Neurologists rated 83% of encounters as ‘satisfied/very satisfied’. EMS clinicians - 90% of encounters ‘satisfied/very satisfied’.	**Destination:** None **Treatment:** Not reported	Not reported	Not reported
**iPad with video capability** Shah et al. 2017 [37]	**Purpose:** Stratify stroke/facilitate care **Diagnostic accuracy:** Not reported **Comparator:** Standard EMS transport (time) **Clinical scales:** Cincinnati Stroke Scale and NIH-8	Not reported	Not reported	**Destination:** Not reported **Treatment:** Not reported	**Door to CT order:** Mean decrease 6 mins (95% CI = 3.6–8.5) **Door to CT study start:** Mean decrease 12 mins (95% CI = 9.4–14.6) **Door-to-CT result:** Mean decrease 12.6 mins (95% CI = 9.7–15.5) **CT order to CT result**: Mean decrease 6.9 mins (95% CI = 4.5–9.3)	Not reported
**Field-Telestroke** Andrefsky et al. 2018 [38]	**Purpose:** Stratify stroke/facilitate care **Diagnostic accuracy:** Not reported **Comparator:** Standard EMS transport (time) **Clinical scale:** None reported	Not reported	Not reported	**Destination:** None **Treatment:** Non-significant increase in thrombolysis (10.6–12.7%)	**Door-to-scan time:** Telestroke (10.7 mins) Standard EMS (34.5 mins) (improvement 23.8 mins) **Door-to-needle time:** Telestroke (41 mins) Standard EMS (50 mins) (improvement 9 mins)	Not reported
**REACHOUT** Hackett et al. 2018 [39]	**Purpose:** Stratify stroke/facilitate care **Diagnostic accuracy:** Not reported **Comparator:** Hospital telestroke (time) **Clinical scale**: None	Not reported	Not reported	**Destination:** Not reported **Treatment:** Not reported	**Door-to-needle time:** Significant median reduction of 26 min with EMS telestroke (median 39.5 mins) compared with hospital based telestroke (median 65.5 mins)	Not reported
**Custom-built system** Johansson et al. 2019 [40] CE Marked	**Purpose:** Stratify stroke/facilitate care **Diagnostic accuracy:** Not assessed **Comparator:** Acceptability / usability of the new telestroke system vs current practice **Clinical scale:** PreHAST and NIHSS	Not reported	4 EMS nurses & 1 remote physician: 2 EMS nurses stated the system was reliable; 3 considered it to be safe. Minor operating interference, physicians’ competence crucial and unclear efficacy emerged from analysis of free text. Remote physician - image quality ‘more than satisfactory’.	**Destination:** Not assessed **Treatment:** Not assessed	3 out 4 of EMS nurses did not believe that the system yielded a more uniform assessment or would reduce time-to-treatment	Not reported

All telemedicine systems included video and audio components, with exception of Stroke Angel in which stroke screening information was collected and transferred from the ambulance to hospital. Earlier studies [26–30] utilised technology with lower resolution and slower transmission speeds than later studies [31–40] employing contemporary technology such as high definition, bi-directional video communication and 4G networks. Systems were either purpose-built or adapted from commercially available technology (e.g. tablet PCs). Most telemedicine systems were in the Beta stage of development, with exception of three Gamma stage systems [30, 33, 34].

The need for EMS clinician training on use of telestroke systems was reported for all but two studies [35, 39]. EMS clinicians were predominately paramedics, with three studies employing EMS nurses (equivalent to paramedics in these countries) [31, 33, 40] and one [28] EMS physicians and paramedics (with the aim of obviating the need for EMS physicians).

Costs were rarely reported, limiting comparison between studies. Where reported [26, 27, 34–36], costs are based on year of publication prices (converted costs were calculated using historical exchange rates but not adjusted for inflation). None of the studies reported on the full range of costs required to implement telestroke (training, unit, operating and maintenance).

A variety of existing and commonly used pre-hospital and hospital-based stroke screening scales were used in conjunction with the telestroke systems (Table 5). Three studies evaluated and used a bespoke telemedicine scale. A 14-item stroke history checklist was developed by experts based on published checklists and recommendations and evaluated for use in conjunction with the ‘peeq-box’ system [28]. The PreSSUB I and II studies [31, 32] developed and evaluated the Unassisted Telestroke Scale; included items were based on existing stroke scales and evaluation of their appropriateness by experts [45, 46].

Data on diagnostic accuracy of telestroke systems were reported in five studies [28, 29, 31, 34, 35]. Pre-hospital stroke diagnosis (versus other neurological/non-neurological diagnoses) using the ‘peeq-box’ telestroke system was comparable to standard EMS transport and hospital confirmed diagnoses of stroke [28]. The Stroke Angel telestroke system, utilising the Los Angeles Pre-hospital Stroke Screen, had only moderate sensitivity (66%) and specificity (62%) for a diagnosis of stroke in the pre-hospital setting [29]. The PreSSUB I study [31] reported an equivalent rate of stroke diagnosis between telestroke and hospital-based clinical assessments (80 and 83% respectively). The InTouch Express telestroke system, using the Cincinnati Pre-hospital Stroke Scale, had equivalent rates of true/false positives for stroke diagnosis compared with standard EMS transport [34]. Finally, a smartphone telestroke system with encrypted software using the National Institute of Health Stroke Scale (NIHSS) reported ‘high’ intra-rater reliability with hospital-based NIHSS assessment [35].

Eleven of 15 studies [26–29, 31–36, 40] evaluated acceptability/usability of telestroke systems from the perspective of EMS clinicians and remote physicians using mixed methods. Results were positive, with studies reporting only minor issues related to connectivity [28, 34] and high levels of satisfaction with systems [26, 27, 29, 31, 33–36], image quality, reliability, usability or perceived safety [26–29, 33–35, 40]. One study reported only 25% of EMS nurses believed telestroke could improve assessments and reduce time-to-treatment due to concerns about clinician ability to use systems and integration into standard care processes [40]. Robust data on patient acceptance was not reported.

Time metrics were reported for 11 of 15 telestroke studies [28–34, 36–39]. Duration of telestroke consultation was reported in three [31, 34, 36]. PreSSUB I [31] consultations were 9 min (IQR 8–13 min). InTouch Xpress [34] consultations were 7.3 and 4.7 min (mean) for thrombolytic and non-thrombolytic patients respectively. Mean duration of NIHSS via the HipaaBridge system was 7.6 min [36].

With the Stroke Angel system, which allows transfer of relevant data to remote clinicians, travel time reduced by 4 min versus standard EMS transport [29]. Call-to-door time increased (2 min) and call-to-scene time matched standard care. The In-Touch Xpress study assessed onset-to-scene time [34] with a non-significant decrease of 18.9 min. Where evaluated, there were no significant differences in time-on-scene [28–30] and scene-to-door time [28, 34] between telestroke and standard EMS transport. PreSSUB II was the only study to assess Call-to-CT time [33], reporting a significant mean reduction of 36.4 min (95% CI = 17.5 to 55.3) with telestroke. Door-to-CT time was improved in four studies [28–30, 37] ranging from 12 min [30] to 24 min [29]. One study utilising IPads [37] reported significantly reduced door-to-CT start (12 min) and result (13 min). Four studies reported improved door-to-needle times [29, 34, 38, 39], two of which statistically significantly [34, 39], ranging from 13 min (InTouch Xpress versus standard EMS transport) [34] to 26 min (REACHOUT versus hospital-based telemedicine) [39]. InTouch Xpress telestroke significantly decreased onset-to-needle time (32 min) [34].

Excluding one study, where suspected stroke patients were taken directly to the nearest specialist centre [35], telestroke studies did not assess impact on EMS clinician decisions as a function of hospital destination (stroke-specialist centre versus non-specialist centre). Impact on IVT rates were assessed in four studies [28–30, 38]; two reported non-significant differences versus standard EMS transport (‘peeq-box’ [28] and Field-Telestroke [38]). Compared with standard EMS transport, the Stroke Angel system elicited significant increases in IVT, with 7% [30] and 5% [29] increases over 9 and 3-year periods respectively. However, neither study adjusted for concurrent increases in the thrombolysis rate. PreSSUB II [33] has yet to report on this.

Few data are available on patient safety outcomes. Stroke Angel [29] reported a 1.5% rate of symptomatic intracerebral bleeding and 11% mortality rate with use of the system. However, a-priori rates were not reported. PreSSUB II [33] reported no telestroke-related adverse events and equivalent mortality outcomes as with standard EMS transport. The InTouch Xpress [34] telestroke system also had equivalent mortality (zero), but a lower complication rate (1 vs 5 respectively), compared with the standard EMS transport group. None of the telestroke studies reported on patients’ functional health outcomes, although PreSSUB II [33] plans to.

## Discussion

Three categories of pre-hospital technologies, with intended use by EMS clinicians to facilitate stroke care, were identified: two direct diagnosis methods (biomarkers and pre-hospital imaging) and one adjunctive technology to facilitate stratification (mobile telemedicine/telestroke). Although telemedicine systems have been in development for some years and are relatively mature, there was little robust evidence of impact on patient outcomes. Biomarker and other diagnostic technologies are at much earlier stages of development.

Blood sampling for biomarkers in the pre-hospital setting appears feasible [41]; however, there is currently no published evidence on diagnostic accuracy and patient outcomes for the studies identified: Helsinki Ultra-acute Stroke Biomarker Study [22] and PRISM [23]. The Helsinki study examines GFAP which appears promising for identifying haemorrhage at an early time point [47–49]; however, this biomarker may not be robust and, as it does not identify small haemorrhages with the same performance as large ones, many not be useful to inform IVT decisions [50]. The other Helsinki biomarker, NR2 peptide, has potential for diagnosing ischaemia but data within the first 6 h is limited [51, 52]. The role of additional biomarkers may be crucial. There is evidence supporting the measurement of purines as an indicator of cerebral ischaemia, where increases corresponded with hypo-perfusion induced by carotid clamping [53] and correlated with greater stroke volumes in the emergency department [42, 54]. However, the applicability of this to the pre-hospital setting is currently unconfirmed. There are many other candidate stroke diagnostic biomarkers [55–65]. Inflammatory and anti-inflammatory cytokines may have utility in diagnosing ischaemia [62–65], but may not be useful in the hyper-acute phase due to their late temporality after stroke onset [63–68]. Validation is challenging due to the various clinical subgroups within the suspected stroke population, wide ranges for normal values and latency of some of these biomarkers, which would also limit their application in hyper-acute diagnoses [57–61, 69]. As isolated biomarkers do not appear to have adequate accuracy for a definitive diagnosis, some evidence suggest a combination of biomarkers, reflecting various stroke-related biological processes, may be optimal [59, 70–73].

A previous review of pre-hospital imaging technologies for stroke diagnosis identified 10 devices in development [74]. However, only two devices fulfilled our review criteria of application during pre-hospital care. We identified a single-centre pre-hospital pilot study of an infrared screening device, reporting moderate sensitivity (~ 71%) and poor specificity (~ 40%) for differentiating haemorrhagic stroke from ischaemia and mimics [24] with diagnostic accuracy influenced by speed of the moving ambulance [24]. No data are available on redirection of patients to stroke-specific centres or patient health outcomes following clinical use of the device. Similar devices exist but have not been assessed in the pre-hospital setting and some not yet in humans [75–78]. ELECTRA-STROKE has yet to report on outcomes. There are alternative electrophysiological devices with intended application to the pre-hospital setting [79–82] but clinical publications are lacking. Other potential pre-hospital imaging technologies include Volumetric Integral Phase Shift Spectroscopy (VIPS) by Cerebrotech Medical Systems, Inc., which has been evaluated in hospital but not the pre-hospital setting. VIPS uses electromagnetic induction to detect ischaemic stroke (including LVO) via hemispheric bioimpedence asymmetry. Evidence of diagnostic accuracy is promising and a new study is ongoing [83, 84]. Similar technologies exist but at an earlier stage of development without pre-hospital data reported [85–93] including magnetic particle imaging [94] ultrasonography [95–101] and accelerometery [102, 103].

The European Academy of Neurology and European Stroke Organization consensus statement for pre-hospital management of stroke did not support routine use of pre-hospital telemedicine for suspected stroke [104]. However, we identified evidence from observational studies and one RCT [26–40] of the safety, feasibility, and potential scalability of telemedicine, with equivalent diagnostic accuracy to hospital-based clinical diagnoses. Telestroke may expedite time-to-treatment by attenuating hospital-based assessment, but studies to date have shown little evidence of more efficient patient redirection to stroke-specific centres and no impact on health outcomes for specific population groups. There are no trial reports of telestroke use to improve the delivery of thrombectomy. Although reported running costs were relatively low, none of the studies reported on the full range of costs required for implementation (training, unit, operating and maintenance) which would inform commissioning decisions. Nevertheless, increased efficiency with telestroke (e.g., 13 min reductions in door-to-needle time [34]) is congruent with mobile stroke units [105], which also lack clear evidence of better patient health outcomes [104]; telestroke technology could be a significantly more cost-effective alternative in systems which do not have specialists present in the ambulance [34]. In conjunction with clinician acceptability for the majority of telestroke systems, the modest goal (stratification) permits feasibility of implementing telestroke technologies in the pre-hospital setting in the near future; however, further studies are still required. Later-stage barriers to implementation should also be addressed in prior development work and monitored with qualitative process evaluations alongside RCTs. One study evaluating the acceptability/usability of a custom-built telestroke system highlighted that EMS clinicians’ views on the potential efficacy of any new system is important for successful deployment [40]. This underscores the need to engage with relevant clinicians in the development and testing of pre-hospital interventions.

In terms of quality, the included pre-hospital imaging and biomarker studies were appropriately designed to assess diagnostic accuracy, with reference standards as comparators. With exception of PRESSUB II [32, 33] and Stroke Angel [29, 30], none of the included telemedicine studies were adequately designed to formally assess diagnostic accuracy, although some confirmed the use of final clinical diagnosis as a comparator [28, 31, 35]. As telemedicine cannot provide a definitive diagnosis, it is more pertinent to assess scale reliability and reductions in time-to-treatment via triage facilitation. The majority of telemedicine studies did assess time metrics and therefore efficacy [28–30, 32, 33, 35, 37–39], but were unblinded observational reports and should be considered as service evaluations rather than definitive evidence. Apart from two telemedicine studies [32, 35], none of the studies reported or planned to report cost-effectiveness. With exception of some large studies, the majority of studies were small, single-centre studies and so lacked robust evidence.

The strengths of this review include a comprehensive structured search strategy and independent assessment at each stage of the review process. However, unpublished data reporting on the efficacy of pre-hospital stroke technology was not included. Additionally, many emerging candidate technologies at earlier research stages were omitted due to a lack of pre-hospital testing, although the intention for most is to conduct pre-hospital trials in the future. The results are limited by the information provided in published reports about the technology, clinical population and reference standard. There were no high quality diagnostic accuracy studies. As a decision support technology, telemedicine could act as a precursor to hospital-based imaging and so will always have limited value when used in isolation. No study combined telestroke with other technologies that may enhance stratification.

In future, efficacy of technologies aiming to provide definitive diagnoses (biomarkers and portable imaging) should be first established in the hospital setting with a clearly stated reference standard (brain imaging and specialist review) as a comparator. Using the most promising technologies, it would then be important to undertake appropriate multi-centre studies comparing standard EMS with a combination of direct stratification (portable imaging and/or biomarkers) and facilitation (telestroke) technologies, as well as additional validated pre-hospital clinical assessment scales. This would establish: 1). efficacy of stroke diagnosis and stratification across different pathways/service configurations; 2). additional value over existing pre-notification systems; 3). impact on service optimisation (particularly, minimisation of secondary transfer for thrombectomy); 4). whether improved process measures (e.g. time metrics and stratification) translate into clinically significant improvements in patient health and quality of life outcomes. Once validated, impact on treatment decisions and patient outcomes can be evaluated. Health economic evaluations would also provide insights into cost-effectiveness to inform decision making by commissioners.

## Conclusions

Although there is clear recognition of the potential value for using emerging technology during the pre-hospital diagnosis or stratification of suspected stroke, a lack of high quality pre-hospital data on biomarkers and portable imaging technologies suggests that further development and validation in the pre-hospital setting is first required. Evaluations of telestroke systems for diagnosis and stratification of stroke indicate they are feasible and safe, but they lack robust evidence for impact on service optimisation and cost-effective patient health outcomes. Multi-centre diagnostic accuracy and clinical utility studies combining these promising direct and adjunctive pre-hospital diagnostic technologies are warranted to inform recommendations on their use. Further development and validation of promising technologies has the potential to revolutionise acute stroke diagnosis and stratification.

## Supplementary information

**Additional file 1.** Search Strategy and Data Extraction Form.

## Data Availability

This is not applicable as no primary data were collected for this review.

## Abbreviations

MT: Mechanical Thrombectomy
LVO: Large Vessel Occlusion
FAST: Face Arm Speech Test
CT: Computed Tomography
MRI: Magnetic Resonance Imaging
MeSH: Medical Subject Headings
EMS: Emergency Medical Services
TIDieR: Template for Intervention Description and Replication
IVT: Intravenous Thrombolysis
mRS: modified Rankin Scale
GFAP: Glial Fibrillary Acidic Protein
NR2: N-Methyl-D-Aspartate Receptor 2
EEG: Electroencephalography
RCT: Randomised Controlled Trial
PC: Personal Computer
NIHSS: National Institute of Health Stroke Scale
VIPS: Volumetric Integral Phase Shift Spectroscopy
