# Supplementary material for: A scoping review of pre-hospital technology to assist ambulance personnel with patient diagnosis or stratification during the emergency assessment of suspected stroke
Source: BMC Emerg Med. 2020 Apr 26;20:30. doi: 10.1186/s12873-020-00323-0 (PMC7183583; doi:10.1186/s12873-020-00323-0)
Supplement: Supplementary file 1 — Additional file 1. Search Strategy and Data Extraction Form. [file 12873_2020_323_MOESM1_ESM.docx]

**Appendix 1: Search Strategy**

Database Search Strategies (restrict to year 2000 for articles and restrict to 2013 for conference abstracts):

**Medline:**

Key Word and MeSH term combination:

Population/ Context

Intervention/ Concept

Outcome

1. exp Stroke/di or Cerebrovascular Disorders/di or exp Brain Ischemia/di or stroke.mp. or CVA.mp. or cerebrovascular accident.mp. or large vessel occlusion.mp.
2. exp Emergency Medical Services/ or Paramedic*.mp. or ambulance.mp. or triage.mp. or prehospital.mp. or pre hospital.mp. or EMS.mp. or emergency medical service*.mp.
3. 1 AND 2
4. exp Biomarkers/ or exp Diagnostic imaging/cl, mt or exp Technology/cl, mt or Biomarker*.mp. or imaging.mp. or technology.mp.
5. exp Telemedicine/cl, mt or exp Remote Consultation/cl, mt or Telemedic*.mp. or telecommunicat*.mp. or teleconsult*.mp. or remote consultation.mp. or telestroke.mp. or telehealth.mp.
6. 4 OR 5
7. exp diagnosis/ OR Diagnos* OR assess* OR identif* OR stratif* or facilit* OR detect*
8. 3 AND 6 AND 7

**Web of Science:**

Topic Search and Web of Science category combo:

Population/ Context

Intervention/ Concept

Outcome

|  | TS = (stroke OR CVA OR cerebrovascular accident OR large vessel occlusion) |
| --- | --- |

1. WC = (emergency medicine OR health care sciences services) OR TS = (Paramedic* OR ambulance OR triage OR prehospital OR pre hospital OR pre-hospital OR EMS OR emergency medical service*)
2. 1 AND 2
3. WC = (engineering biomedical OR medical laboratory technology OR neuroimaging OR radiology nuclear medicine medical imaging OR biotechnology applied microbiology) OR TS = (Biomarker* OR imaging OR technology)
4. WC = (telecommunications OR medical informatics) OR TS = (Telemedic* OR telecommunicat* OR teleconsult* OR remote consultation OR telestroke OR telehealth)
5. 4 OR 5
6. TS = (Diagnos* OR assess* OR identif* OR stratif* or facilit* OR detect*)
7. 3 AND 6 AND 7

**PubMed:**

Keyword and MeSH combo:

Population/ Context

Intervention/ Concept

Outcome

|  | 1. "Brain Ischemia/diagnosis"[Mesh] OR "Cerebrovascular Disorders/diagnosis"[Mesh] OR "Stroke/diagnosis"[Mesh] OR “stroke” OR “CVA” OR “cerebrovascular accident” OR “large vessel occlusion” |
| --- | --- |

1. "Emergency Medical Services"[Mesh] OR “Paramedic” OR “ambulance” OR “triage” OR “prehospital” OR “pre hospital” OR “pre-hospital” OR “EMS” OR “emergency medical service”
2. ("Brain Ischemia/diagnosis"[Mesh] OR "Cerebrovascular Disorders/diagnosis"[Mesh] OR "Stroke/diagnosis"[Mesh] OR “stroke” OR “CVA” OR “cerebrovascular accident” OR “large vessel occlusion”) AND ("Emergency Medical Services"[Mesh] OR “Paramedic” OR “ambulance” OR “triage” OR “prehospital” OR “pre hospital” OR “pre-hospital” OR “EMS” OR “emergency medical service”)
3. "Technology/classification"[Mesh] OR "Technology/diagnosis"[Mesh] OR "Technology/methods"[Mesh] OR "diagnostic imaging"[Mesh] OR "Biomarkers"[Mesh] OR “biomarker” OR “imaging” OR “technology”

|  | 1. "Telemedicine/classification"[Mesh] OR "Telemedicine/methods"[Mesh] OR "Remote Consultation/classification"[Mesh] OR "Remote Consultation/methods"[Mesh] OR “Telemedicine” OR “telecommunication” OR “teleconsultation” OR “remote consultation” OR “telestroke” OR “telehealth” |
| --- | --- |

1. ("Telemedicine/classification"[Mesh] OR "Telemedicine/methods"[Mesh] OR "Remote Consultation/classification"[Mesh] OR "Remote Consultation/methods"[Mesh] OR “Telemedicine” OR “telecommunication” OR “teleconsultation” OR “remote consultation” OR “telestroke” OR “telehealth” OR "Technology/classification"[Mesh] OR "Technology/diagnosis"[Mesh] OR "Technology/methods"[Mesh] OR "diagnostic imaging"[Mesh] OR "Biomarkers"[Mesh] OR “biomarker” OR “imaging” OR “technology”)
2. **"diagnosis"[Mesh] OR “Diagnosis” OR “diagnose” OR “assess” OR ”identify” OR “identification” OR “stratify” OR “stratification” OR “facilitate” OR “detect” OR “detection”**
3. ("Brain Ischemia/diagnosis"[Mesh] OR "Stroke/diagnosis"[Mesh] OR “stroke” OR “CVA” OR “cerebrovascular accident” OR “large vessel occlusion”) AND ("Emergency Medical Services"[Mesh] OR “Paramedic” OR “ambulance” OR “triage” OR “prehospital” OR “pre hospital” OR “pre-hospital” OR “EMS” OR “emergency medical service”) AND ("Telemedicine/classification"[Mesh] OR "Telemedicine/methods"[Mesh] OR "Remote Consultation/classification"[Mesh] OR "Remote Consultation/methods"[Mesh] OR “Telemedicine” OR “telecommunication” OR “teleconsultation” OR “remote consultation” OR “telestroke” OR “telehealth” OR "Technology/classification"[Mesh] OR "Technology/diagnosis"[Mesh] OR "Technology/methods"[Mesh] OR "diagnostic imaging"[Mesh] OR "Biomarkers"[Mesh] OR “biomarker” OR “imaging” OR “technology”) AND ("diagnosis"[Mesh] OR “**Diagnosis” OR “diagnose” OR “assess” OR ”identify” OR “identification” OR “stratify” OR “stratification” OR “facilitate” OR “detect” OR “detection”)**

**Embase:**

Keyword and MeSH combo:

Population/ Context

Intervention/ Concept

Outcome

1. Stroke OR CVA OR cerebrovascular accident OR large vessel occlusion OR exp cerebrovascular accident/di [Diagnosis]
2. Paramedic* OR ambulance OR triage OR prehospital OR pre hospital OR pre-hospital OR EMS OR emergency medical service* OR emergency care/ OR *emergency health service/
3. 1 AND 2
4. Biomarker* OR imaging OR technology OR exp diagnostic imaging/ OR exp biological marker/ OR medical technology/ OR biotechnology/
5. Telemedic* OR telecommunicat* OR teleconsult* OR remote consultation OR telestroke OR telehealth OR exp teleconsultation/ or exp telediagnosis/ or exp telepathology/ or exp teleradiology/
6. 4 OR 5
7. Diagnos* OR assess* OR identif* OR stratif* or facilit* OR detect* OR diagnostic procedure/ OR diagnosis/ OR clinical classification/
8. 3 AND 6 AND 7

**Clinical Trials Search Strategy**

Topic: Stroke OR cerebrovascular accident

(prehospital OR paramedic OR emergency) AND (technology OR telecommunication OR telemedic* OR imaging OR biomarker)

**Appendix 2: Data Extraction Form**

Study ID:

| **GENERAL INFORMATION** | | | | | | | |
| --- | --- | --- | --- | --- | --- | --- | --- |
| **Date of data extraction:** | | | | |  | | |
| **Data extracted by:** | | | | |  | | |
| **Full reference:** | | | | |  | | |
| **Email address for corresponding author:** | | | | |  | | |
| **Country of origin:** | | | | |  | | |
| **Year of Publication:** | | | | |  | | |
| **Setting:** | | | | |  | | |
| **Source of funding:** | | | | |  | | |
| **STUDY CHARACTERISTICS** | | | | | | | |
| **Study design:** | ***Quantitative***   - Prospective - Retrospective - Mixed methods - **AND** - Single centre - Multicentre   **AND:**   - RCT - Diagnostic accuracy - Cohort study - Development - Feasibility - Pilot test - Non-randomised controlled trial - Case study - Case series - Other (please state): | | | | | ***Qualitative***   - Please describe: | ***Mixed methods***   - Please describe: |
| **Objectives:** | | | | | | | |
| **Inclusion criteria:** | | |  | | | | |
| **Exclusion criteria:** | | |  | | | | |
| **Study sample size** | | | | ***Informed by power calculation***: Yes / No / N/A | | | |
| **No in intervention group** | | | | n = | | | |
| **No in comparison group** | | | | n = | | | |
| **Comparator:** | | | |  | | | |
| **Follow-up time points** | | | | Time points: Not applicable | | | |
| **Loss to follow-up** | | n = % = not reported | | | | | |

| **MODIFIED TIDIER ITEMS** |
| --- |

| **Category** | **Subcategory and code** |
| --- | --- |
| **1. Type of technology, stage of development and degree of portability** | **1.1 Type of technology:**  1.1A Prehospital Imaging  1.1A1 Magnetic Particle Imaging  1.1A2 Magnetic Resonance Imaging  1.1A3 Magnetic Particle Imaging (e.g. MIPS)  1.1A4 Electrophysiology (e.g. EEG)  1.1A5 Electromechanical Physiology (e.g. Accelerometery)  1.1A6 Electrical Impedence Spectroscopy  1.1A7 Ionising Electromagentic Tomography (e.g. CT)  1.1A8 Electromagnetic Spectroscopy (Light)  1.1AA Technical Specification (resolution)  1.1AA1 Low Resolution Technique  1.1AA2 Moderate Resolution Technique  1.1AA3 High Resolution Technique  1.1B Telestroke  1.1B1 Wearable/portable – Data Transfer  1.1B2 Wearable/portable – 2 way audio communication  1.1B3 Wearable/portable – 1-way video communication  1.1B4 Wearable/portable – 2-way video communication  1.1B5 Ambulance based: Data Transfer  1.1B6 Ambulance based: 2-way audio communication  1.1B7 Ambulance based: 1-way video communication  1.1B8 Ambulance based: 2-way video communication  1.1BA Technical Specification (resolution)  1.1BA1 Low Resolution (VGA – 640x480)  1.1BA2 Moderate Resolution (SD – 720x480)  1.1BA3 High Resolution (HD-FHD: 1280-1920x720-1080)  1.1BB Technical Specification (speed)  1.1BB1 Slow Data Transmission (2G - <100kb/s – 300kb/s)  1.1BB2 Moderate Data Transmission (3G – 300kb/s - 4200kb/s)  1.1BB3 Fast Data Transmission (4G – 150000kb/s - 979000kb/s)  1.1C Molecular Biomarkers  1.1C1 Proteins  1.1C2 Enzymes  1.1C3 Neurotransmitters  1.1C4 Hormones  1.1C5 Cells  1.1C6 Lipids  1.1C7 Genes  1.1C8 Antibodies  1.1D: Other Details |
|  | **1.2 Stage of technology development:**  1.2A Alpha (i.e. initial prototype stage)  1.2B Beta (i.e. later iteration of the prototype, but not the final product)  1.2C Gamma (i.e. finalised product available for wider clinical use) |
|  | **1.3 Portability:**  1.3A Highly portable (hand-held)  1.3B Portable (with use of vehicle)  1.3C Not portable |
| **2. Goal of the technology** | **2.1 Purpose:**  2.1A Stroke  2.1A1 Stratification/facilitation of diagnosis  2.2A2 Diagnosis  2.1B Haemorrhagic stroke  2.1B1 Stratification/facilitation of diagnosis  2.1B2 Diagnosis  2.1C Ischaemic stroke  2.1C1 Stratification/facilitation of diagnosis  2.2C2 Diagnosis  2.1D: Ischaemic stroke: SVO  2.2D1 Stratification/facilitation of diagnosis  2.2D2 Diagnosis  2.1E: Ischaemic stroke: LVO  2.2E1 Stratification/facilitation of diagnosis  2.2E2 Diagnosis  2.1F Mimic/TIA  2.1F1 Stratification/facilitation of diagnosis  2.1F2 Diagnosis  2.1G Other (state details) |
| **3. Users of the technology** | **3.1 Expertise/background and specific training given (if applicable) of the intended user(s):**  3.1A Qualified Paramedic  3.1B Other ambulance staff (e.g. technicians)  3.1C Further Details |
|  | **3.2 Output interpreted by one individual, or need for confirmation by an**  **external interpreter**  3.2A One individual  3.2B Confirmation by external operator |
|  | **3.3 Training needed to use technology:**  3.3A Yes – Formal Training (state details)  3.3B Yes – Informal Training (state details)  3.3C No (state details) |
| **4. Procedures and processes for implementing the technology** | **4.1 Does the result need clinical interpretation?**  4.1A Yes (state details)  4.1B No (state details) |
| **5. Location in which technology can be used** | **5.1 Point(s) on the care pathway that technology can be used**  5.1A On scene  5.1B In ambulance (stationary)  5.1C In ambulance (during transit) |
| **6. Time needed to use the technology** | **6.1 Time to acquire results from initiation of device to output (in minutes)** |
| **7. Strategies employed to ensure the technology was used as intended** | **7.1 How was this assessed?**  7.1A Fidelity assessment (video) (state assessment result)  7.1B Fidelity assessment (independent observer) (state assessment result)  7.1C Inter-rater reliability (state assessment result)  7.1D Intra-rater reliability (state assessment result)  7.1E Other (state details) (state assessment result) |
| **8. Efficacy and effectiveness of the technology** | **8.1 Stratification: evidence for diagnostic accuracy of technology:**  8.1A Sensitivity (state value(s))  8.1B Specificity (state value(s))  8.1C Likelihood ratio (state value(s))  8.1D Diagnostic odds ratio (state value(s))  8.1E Area under the ROC curve (state value(s))  8.1F Positive predictive value (state value(s))  8.1G Negative predictive value (state value(s))  8.1H Accuracy (state value(s))  8.1I Adjunctive clinical scale used (state details) |
|  | **8.2 Impact on process outcomes**  8.2A Onset to scene time (state change)  8.2B On scene time (state change)  8.2B Scene to door time (state change)  8.2C Door to needle time (state change)  8.2D Onset to door time (state change)  8.2E Door to scan time (state change)  8.2F Scan to needle time (state change)  8.2G Onset to treatment time (state change)  8.2H Onset to puncture time (state change)  8.2I Secondary transfer time (state change)  8.2J Door to puncture time (state change)  8.2K Travel Time (state change)  8.2J Secondary Transfer (state change in time or necessity) |
|  | **8.3 Impact on clinical outcomes:**  8.3A Independence (state values and measure used, i.e. NIHSS/mRS)  8.3B Mortality (state values)  8.3C Complications (state values) |
|  | **8.4 Impact on pre-hospital care provider decisions**  8.4A Direct transfer to nearest HASU (state details)  8.4B Direct transfer to a comprehensive stroke centre (state details)  8.4C Secondary transfers (state details)  8.4F None |
| **9. Acceptability, usability and feasibility** | **9.1 Physical invasiveness:**  9.1A Invasive (penetration/ breaking of skin or entrance into a body cavity)  9.1B Minimally-invasive (indirect observation of internal areas of the body)  9.1C Non-invasive (no penetration/breaking of skin or entrance into a body cavity) |
|  | **9.2 Acceptability to patients**  9.2A Assessed with patient interviews  9.2B Assessed using questionnaires  9.2C Assessed using group interviews/focus groups  9.2D Assessed using other methods  9.2E Not assessed |
|  | **9.3 Usability (clinicians)**  9.3A Assessed with interviews  9.3B Assessed using questionnaires  9.3C Assessed using group interviews/focus groups  9.3D Assessed using other methods  9.3E Not assessed |
|  | **9.4 Feasibility / implementation barriers:**  9.4A High cost  9.4B Time efficiency unacceptable  9.4C Limited availability  9.4D Lack of user confidence in technology  9.4E Lack of wider stakeholder support  9.4F Lack of clinical service endorsement  9.4G Other (state details) |
| **10. Cost** | **10.1 Cost of technology development (and implementation):**  Actual or predicted cost in pounds Sterling |
|  | **10.2 Cost-effectiveness in QALYs (or other cost-effectiveness measure)** |
| **11. Safety Legislation** | **11.1 Requirements:**  11.1A Radio frequencies met (specific absorption rate: IEEE safety limit of 2W/kg)  11.1B Ionising radiation met (radiation safety limit 1mW/cm2)  11.1C Not met |
|  | **11.2 Approved for use by regulatory body (FDA; NICE; MHRA; other)**  11.2A Yes (Specify approval provider)  11.2B No |

NA – Not Applicable, NR – Not Reported
